# Interventions to Assist Health Consumers to Find Reliable Online Health Information: A Comprehensive Review

**DOI:** 10.1371/journal.pone.0094186

**Published:** 2014-04-07

**Authors:** Kenneth Lee, Kreshnik Hoti, Jeffery D. Hughes, Lynne M. Emmerton

**Affiliations:** School of Pharmacy, Curtin University, Perth, Western Australia, Australia; University of Louisville, United States of America

## Abstract

**Background:**

Health information on the Internet is ubiquitous, and its use by health consumers prevalent. Finding and understanding relevant online health information, and determining content reliability, pose real challenges for many health consumers.

**Purpose:**

To identify the types of interventions that have been implemented to assist health consumers to find reliable online health information, and where possible, describe and compare the types of outcomes studied.

**Data Sources:**

PubMed, PsycINFO, CINAHL Plus and Cochrane Library databases; WorldCat and Scirus ‘gray literature’ search engines; and manual review of reference lists of selected publications.

**Study Selection:**

Publications were selected by firstly screening title, abstract, and then full text.

**Data Extraction:**

Seven publications met the inclusion criteria, and were summarized in a data extraction form. The form incorporated the PICOS (Population Intervention Comparators Outcomes and Study Design) Model. Two eligible gray literature papers were also reported.

**Data Synthesis:**

Relevant data from included studies were tabulated to enable descriptive comparison. A brief critique of each study was included in the tables. This review was unable to follow systematic review methods due to the paucity of research and humanistic interventions reported.

**Limitations:**

While extensive, the gray literature search may have had limited reach in some countries. The paucity of research on this topic limits conclusions that may be drawn.

**Conclusions:**

The few eligible studies predominantly adopted a didactic approach to assisting health consumers, whereby consumers were either taught how to find credible websites, or how to use the Internet. Common types of outcomes studied include knowledge and skills pertaining to Internet use and searching for reliable health information. These outcomes were predominantly self-assessed by participants. There is potential for further research to explore other avenues for assisting health consumers to find reliable online health information, and to assess outcomes via objective measures.

## Introduction

There is a growing body of evidence that health consumers increasingly rely on the Internet for health information [1]–[8]. This increase is largely due to 1) the immense abundance of online information [9], [10], decision aids [11] and Web 2.0 health applications [10], [12], [13], 2) the increasing prevalence of chronic disease in society [14], and 3) the pervasiveness and accessibility of Information Technology in our daily lives [15].

While the role of health professionals in advising and assisting health consumers with decision making is undisputed [1], [16], [17], it is unrealistic, in terms of the health professional's time and ability, to control out-of-consultation behavior of consumers, and to expect consumers to rely entirely on written and verbal information provided by their regular practitioner(s). Indeed, health consumers empowered by information-seeking online may be more engaged in the management of their health conditions, and form more productive relationships with their healthcare practitioners [18].

In order to effectively engage in self-management, health consumers must be able to effectively find, understand and utilize relevant health information [19]–[21]. At the same time, health consumers must also be able to discern reliable from less-reliable information. Collectively, these abilities and skills constitute the definition of an individual's health literacy [19]–[21]. The literature suggests that many health consumers have low levels of health literacy [21]–[26] which hinders their ability to effectively find, understand and use reliable health information to assist them manage their health conditions. As use of the Internet requires technological knowledge and skills [9], finding health information online potentially becomes an even greater burden on consumers with poor computer literacy as well as low levels of health literacy.

### Rationale and Objectives

Given the prevalence of chronic diseases in society and the important role self-management plays in chronic disease management, there appears to be a need for initiatives to assist health consumers to develop their capacity to find reliable health information on the Internet, thereby potentially contributing to improved levels of health literacy. Our comprehensive review follows a systematic review by Car *et al.* [9] that examined the effects of online health literacy training interventions on health outcomes. We intended to focus on the gambit of interventions (face-to-face, online or via other means) implemented by researchers to assist adult health consumers to autonomously find reliable online information about chronic health conditions, and where possible, their outcome measures. This includes, but is not limited to, training interventions.

As the focus of our review is on identifying and descriptively comparing humanistic interventions, as opposed to evaluating formal clinical trials, assessment of the quality of each study (as would be performed in a systematic review) was not formally conducted. Nevertheless, we adopted a systematic approach to the identification and selection of relevant studies, and applied methodological [27] and reporting [28] standards for the conduct of systematic reviews where practical.

## Methods

The method was guided by the Methodological Standards for the Conduct of Cochrane Intervention Reviews [27], where relevant, recognizing the humanistic nature of the interventions of interest.

### Eligibility Criteria

The eligibility criteria for published studies were developed via consultation between all authors of this review, and were:

1. Participants: at least 18 years of age
2. Intervention: any approach where the intention, primary or other, was to assist health consumers in autonomously finding existing reliable online information related to chronic health conditions
3. Language: publications in English.

Publications were included if the study, or a portion of the study, met all of the above criteria. Studies that met criteria 2 and 3, but included some participants less than 18 years of age, were considered relevant. Studies were excluded if the primary intervention involved the *development* of new online health information material, such as producing easy-to-understand material for the purpose of assisting people with low health literacy. Studies were also excluded where assistance was provided to help participants use a particular existing online health website. While the reporting of outcomes for studies is a critical component to evaluating quality, and is desirable, studies that did not report outcomes were not excluded from this review as the primary objective of this review was to identify the types of interventions that have been implemented.

### Information Sources

Four health-related databases and two popular search engines for ‘gray literature’ were utilized for this review. While the term ‘gray literature’ is poorly defined in literature, we defined it as studies published in a format other than academic journals. Reference lists of selected publications were also searched, and authors were contacted where clarification or more information was needed for data analysis.

The databases used were PubMed, PsycINFO, CINAHL Plus and Cochrane Library. Gray literature was searched via WorldCat and Scirus. No limits were applied to dates, although it was expected that most research in this field would be recent due to the relative recency of the Internet and Information Technologies. The last search was conducted on 11 February 2013.

### Search

Search terms and search strings were agreed upon by all authors of this review, and further refined by the first author, KL, after pilot searching various permutations of search terms in PubMed and CINAHL Plus. To optimize efficiency and minimize the volume of irrelevant publications returned, a single search string was used for all databases and gray literature search engines. A second search, using MeSH terms, was conducted on the four databases. This is because MeSH terms are manually assigned after a study has been published; thus, a search using MeSH terms could potentially produced different results. Manual searching of reference lists from selected publications were subsequently conducted to ensure a more comprehensive search.

The following search terms were used to search all databases and gray literature search engines: consumer*; patient*; find*; search*; navigat*; seek*; access*; retriev*; locat*; identify*; “health literacy”; informat*; internet; online; web*.

An example of a search string that was used for this review is:

((consumer* or patient*)) AND ((find* OR search* OR navigat* OR seek* OR access* OR retriev* OR locat* OR identif*)) AND “health literacy” AND informat* AND ((internet or online or web*))

### Study Selection, Data Collection Process and Data Items

After retrieval of publications from the aforementioned databases and search engines, duplicate publications were removed.

A stepwise approach of selecting relevant publications firstly via title, then abstract, then full text was used, adapting relevant sections of the PRISMA flow diagram for reporting of systematic reviews [28]. In this approach, titles of publications were firstly screened for relevance to the review, with relevant publications retained. Where relevance was unclear from examination of title alone, the abstract was then scanned and irrelevant publications were subsequently removed. Publications in which both title and abstract were examined, and relevance to the review was still unclear, were retained for full-text review. Reference lists of selected publications were manually searched for further relevant sources.

Eligibility of publications was further ascertained by full-text review. Authors were contacted for supplementary information where necessary.

A data extraction form was developed to assist data collection and subsequent analysis. This form was developed and piloted by KL, and reviewed by LE. All elements of the PICOS model [28] were incorporated into the form. As few relevant papers were anticipated, trial of the form was not considered necessary, although refinements were incorporated during data extraction. KL extracted data from the included studies, and LE, confirmed the extracted data. Consensus was reached on all points via discussion between KL and LE.

As this review primarily focuses on identifying types of interventions with a view to also identifying types of outcomes where possible, as opposed to examining the effects of these interventions on outcomes, risk of bias in individual studies and across studies was not assessed. It was anticipated that little methodological comparison would be viable.

## Results

### Study Selection

**Figure 1:**
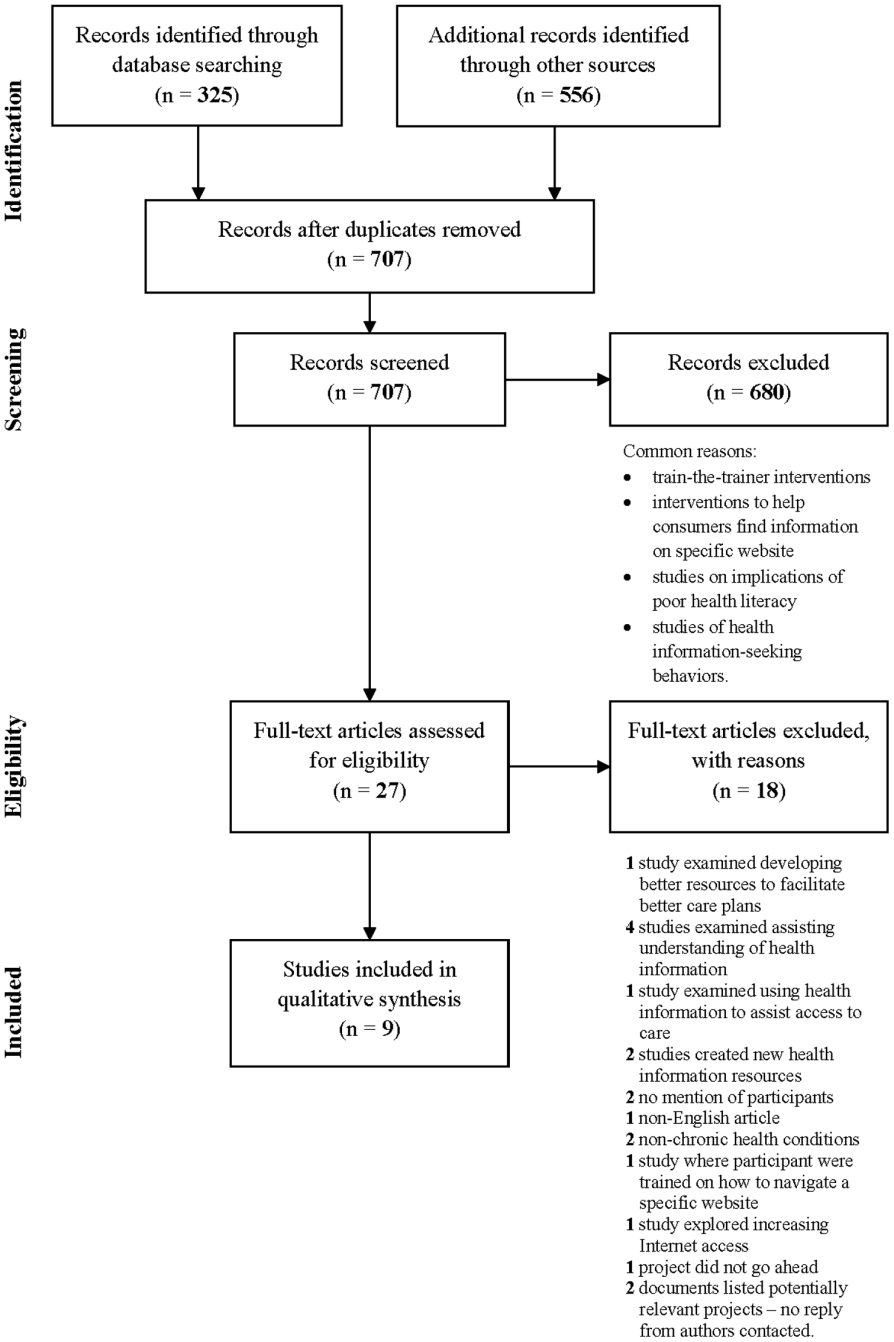
Applies principles of the PRISMA Flow diagram template and outlines the process used to identify, screen, select, and analyse studies for this comprehensive review.

The literature search identified 707 publications after duplicates were removed (Figure 1). Of these, 680 publications were excluded through the screening process (via their titles and abstracts). Common reasons for exclusion were that the publications reported train-the-trainer interventions with no follow-up of the trainers subsequently coaching health consumers, interventions to help consumers find information on a particular website, studies about the various implications of poor health literacy, and exploratory studies of health information-seeking behaviors.

A total of 27 publications were reviewed in full to assess their eligibility for this review. To further assist in assessing eligibility of publications, 14 authors were contacted for supplementary information, with four replies. Overall, seven journal publications and two gray literature reports were deemed eligible for synthesis and analysis. Due to missing data in the gray literature reports, they are described in a separate section from the published studies.

### Study Characteristics

Of the seven published studies [29]–[35], two [29], [32] were randomized controlled trials, and the remaining five [30], [31], [33]–[35] were uncontrolled, single-group trials (Table 1). The number of participants for one study [31] was unknown, while the number of participants for the remaining six studies ranged from 60 to 448. A convenience sampling method was used in all studies. Of the six studies conducted in the United States [29], [31]–[35], three [31], [33], [34] were located in regional or rural parts of the United States. The remaining study [30] was conducted in metropolitan Melbourne, Australia.

**Table 1:** Summary of Interventions, Outcomes, and Brief Critique – Published Studies.

Studies	Sample characteristics	Intervention	Intervention – Style/Content	Outcome(s)	Critique
Carter, Nunlee-Bland and Callender, 2011 [29]	26 African-American adults with Type 2 diabetes, a reading level of at least eighth grade, residing in Washington DC	Online portal with diabetes education modules and communication with a nurse via video conferencing	***Style***: Participants had access to information and links to relevant resources via an online portal. Each participant's self-management plan was developed and constantly reviewed by a telehealth nurse via videoconferencing, guided by results from participants' biometric self-monitoring. *Content*: 1. A culturally-tailored self-management module based on each participant's health records and in discussion with a telehealth nurse. 2. A health education module containing culturally-tailored resources and links to online health websites. 3. A social networking module to encourage participants to ask questions, and share coping strategies and preferred resources.	***Knowledge***: Significant increase in participants' reported knowledge of diabetes and adherence to diabetes management practices (*p*<0.05).	***Strengths***: Randomized-controlled trial; stringent inclusion criteria; baseline and post-intervention assessment; objective measures (biometric/biochemical markers). ***Weaknesses***: Arguably high rate of attrition: 74 participants recruited, 47 completed the study; intervention potentially onerous; no indication of the source of attrition; however, all results from study were from the 47 participants; convenience sample; non-validated instrument; predominantly female population; biometric self-monitoring could have driven other self-care and knowledge-seeking behavior.
Gray, Elliott and Wale, 2012 [30]	Predominantly: Australian (64.6%), 45–64 years old (58.3%), tertiary educated (83.5%)	Interactive workshop	***Style***: Combination of didactic instruction and activities to be completed within a single two-hour workshop. ***Content***: How to judge reliability of health information, credibility of health websites, quality of research; awareness of credible health search engines; access to high quality clinical evidence; and interactive/Web 2.0 health technologies.	***Knowledge***: 60% strongly agreed and 37% agreed that “This workshop improved my knowledge about evidence-based health information”. ***Skills***: 57% strongly agreed and 42% agreed that “This workshop improved my skills with finding and using evidence-based health information”. ***Attitudes***: 78% agreed that the workshop improved their attitude towards evidence-based health information. ***Behavior***: 53% strongly agreed and 41% agreed that the workshop would change the way they looked for and used health information in the future.	***Strengths***: Validated instrument; thorough examination of association between various factors and results (Mann-Whitney U-test and Kruskal-Wallis test); Pre/Post-test. ***Weaknesses***: Small sample size: 89 usable responses from ∼100 surveyed; no control group; self-rated responses; convenience sample.
Gross, Famiglio and Babish, 2007 [31]	Senior citizens residing in a predominantly rural area of North-Eastern Pennsylvania, predominantly aged 65+ years (75%) with at least secondary education (85%)	Stroke education program - two versions of the same program were available: an in-person workshop delivered at 25 centres and an online workshop	***Style***: In-person, unclear – presumed a didactic approach with associated handout of the single, one-hour PowerPoint presentation, a bookmark containing a list of trusted stroke websites and contact information, and a list of trusted health websites. Online – an online version of the in-person PowerPoint presentation. ***Content***: Information on how to prevent, recognize, react and recover from a stroke, as well as information on how to find trusted stroke information on the Internet.	***Knowledge*** *:* General improvement in the areas related to knowledge of Internet resources from baseline.	***Strengths***: Acknowledged contribution of inadequate health literacy to ineffective use of information; Pre-/Post-test; objective test to assess ‘mastery of program’. ***Weaknesses***: Unknown sample size; convenience sample; statistics only used to describe demographics of participants, not for outcomes; no test for statistical significance; no control group; test not validated; unclear procedure around education of participants in finding trusted websites, presumed to involve the neurologist, instructional designer, and librarian who designed the blended education program.
Kalichman, Cherry, Cain, Pope, Kalichman, Eaton, *et al.*, 2006 [32]	448 HIV positive people residing in metropolitan Atlanta, Georgia, mean age 42.5 years, mean 12 years of education, predominantly male (74%) and African-American (89%)	Eight interactive workshops with group activities	***Style***: 120-minute group sessions held twice-weekly for four weeks. In these sessions, participants were encouraged to discuss issues with the group. These group discussions were interspersed with didactic instruction. ***Content***: Exploration of different Web browsers and their functions, how to evaluate the quality of information obtained on the Internet, and how to integrate information found on the Internet with individuals' health care regimen.	***Behavior***: Significantly greater self-efficacy for health information use compared to control group (*p*<0.05).	***Strengths***: Large sample size; stringent screening procedure; randomized-controlled trial; instruments adapted from previously used instruments; multiple time points measured – baseline, 3, 6, 9 months; thorough examination of association between various factors and results (sound statistical procedure and data analysis). ***Weaknesses***: convenience sampling; participants were excluded if they had low reading literacy levels and/or high-Internet usage; control group could be considered an intervention; control group was educated on HIV-related information, whereas intervention group was taught information-evaluation skills.
Kurtz-Rossi and Duguay, 2010 [33]	Students from middle school, high school and an adult learning centre ≤25 years old, residing in rural Maine	School curriculum and community outreach	***Style***: Health literacy curriculum developed into five one-hour lessons with activities, with the final lesson designed as a community outreach activity, whereby students shared what they learnt with either an older family member or a senior in the community. ***Content***: Online health information searching activities, application of a quality health information checklist, evaluation of reliability of websites, and project-based learning in the form of a community outreach activity.	***Knowledge*** *:* 80% agreed that “I am more aware of reliable health information websites.” ***Behavior***: Confidence in ability to evaluate health information that respondents found on the Internet - change from 18% to 48% of respondents from pre to post-intervention.	***Strengths***: Health literacy expert involved in curriculum design; Pre/Post test. ***Weaknesses***: Sample size unclear, but 121 completed post-survey; instrument not validated; no measurement of health literacy, despite health literacy as the focus of the intervention; inconsistent administration of intervention – teachers were allowed to release material at their own pace, potentially not matched to the pace required by participants; no statistical significance testing reported; convenience sample; self-rated outcomes – no objective measures; no control group.
Susic, 2009 [34]	60 senior citizens residing in regional Louisiana, mean age 70 years, predominantly female (79%)	Interactive workshop	***Style***: Combination of didactic instruction and hands-on computer activities. Participants were also given handouts and information packages for further learning. ***Content***: How to navigate the NIHSeniorHealth website; Trainer's Toolkit from NIHSeniorHealth website (Module 2 lesson plan); list of ‘Senior Health Websites You Can Trust’.	***Skill***: More than 80% of the participants could search health databases without assistance post-intervention (no comparison of baseline). Measured by asking participants to find answers to two questions about a given health condition. ***Behavior***: Majority of participants interviewed (n = ?) reported still using the NIHSeniorHealth website to find health information at six months.	***Strengths***: Intervention based on material from NIHSeniorHealth; objective measure for outcomes – participants were to find answers to two questions. ***Weaknesses***: Convenience sampling; poor display of statistics; no control; no pre-/post-test mentioned.
Xie, 2011 [35]	111 older adults, urban area of Maryland, mean age 70.4 years, predominantly female (71%), African-American (66%) with at least secondary education (95%) and economically disadvantaged	Interactive workshops with group activities (Learning framework: collaborative learning)	***Style***: Eight 120-minute group sessions where participants were encouraged to discuss issues, pose real-life questions, and reflect together. These group sessions were interspersed with didactic instruction, but the emphasis was on group collaboration. ***Content***: Information about the NIHSeniorHealth and MedlinePlus website, and basic information about the Internet.	***Knowledge and Skill***: Statistically significant improvement from pre- to post-intervention in general computer/Web knowledge and skills, and in eHealth literacy (*p* values<0.001).	***Strengths***: Sound coverage of learning theories; inclusion criteria clearly reported; comprehensive and validated measures/scales adapted from literature; intervention based on NIHSeniorHealth website; robust analysis with effect sizes; strong pre-/post-test analysis, despite absence of a control group. ***Weaknesses***: Convenience sampling; eHealth Literacy was measured via the eHealth Literacy Scale (eHEALS) [46]; however, this is a scale that assesses perceived eHealth skills [46].

The interventions for three [30], [31], [34] of the seven studies were administered in a single session. The total duration of studies with multiple-session interventions ranged from three weeks to nine months.

Regarding instruments used to measure outcomes, three studies [30], [32], [35] used adapted instruments and one study [29] utilized standard instruments for measuring biochemical and biometric markers of health outcomes in people with diabetes. In terms of the participant outcomes measured, one study [29] indirectly measured health outcomes, while the remaining six studies measured one or more humanistic indicators of health information knowledge, skills and behavior, for which, measurements are not directly comparable. Demographic data for participants were reported in varying degrees for six studies [29]–[32], [34], [35]. Common demographic variables were age [29], [32], [34], [35], gender [29], [30], [32], annual income [29], [30], [32], [35], and level or years of education [29]–[32], [35].

### Design of Interventions

#### Interactive workshops

Interactive workshops featured as the main intervention in five [30]–[32], [34], [35] of the seven published studies. The style of the interactive workshop for one study [31] was unclear, however, four studies [30], [32], [34], [35] used a combination of a didactic approach to teaching and hands-on activities that involved participants searching for health information online. In most of the studies, participants were issued a copy of the presentation slides, as well as lists of credible health websites. Further, discussion amongst participants was encouraged during the workshops in three studies [30], [32], [35]. In particular, collaborative learning was emphasized in one [35] of the studies. Although the intervention for this study included a didactic component, the workshop trainer encouraged interaction between participants. In another study [31], a website hosted the online version of the in-person interactive workshop. This online version used the same content as the in-person workshop.

Common workshop topics included how to judge reliability of health information and credibility of health websites, and awareness of credible websites. Two studies [34], [35] used content from *The Trainer's Toolkit* from the NIHSeniorHealth website (http://nihseniorhealth.gov/toolkit/toolkit.html) as the foundation of their workshops. Participants in one study [31] were provided information on their medical condition, in addition to being educated on how to find credible health information online.

#### Health literacy curriculum and community outreach

In one study [33], a health literacy curriculum was designed and trialed in two middle schools, two high schools and one adult education program, delivered by teachers and librarians. The curriculum was designed as five one-hour lessons with activities, although teachers and librarians were allowed to deliver each lesson at their own pace. As a result, the duration of the intervention ranged from three weeks to three months. In the final lesson, participating students shared what they had learnt with seniors in their community. In this way, seniors were taught by participating students how to find health information online.

The curriculum established that health information is commonly organized via disease type and population, and participants practiced search techniques. Students were also given a checklist adapted from the QUICK website (http://www.asparis.net/lowerschool/quickreview/www.quick.org.uk/menu.htm) for evaluating the credibility and reliability of online health information.

#### Online portal with support via videoconferencing

One study [29] took an arguably more holistic approach, in the development of an online portal to house three modules: self-management, health education, and social networking. The self-management module provided a space for each participant to access their individualized care plans. These plans were reviewed during the bi-weekly videoconferencing with a telehealth nurse, and were amended, if needed, by the participant's physician. During these videoconferences, participants were also encouraged to raise questions with the telehealth nurse. The health education module consisted of age and culturally-appropriate educational videos and links to various health-related websites. The social networking module provided a space for participants in the study to interact with one another, and participants were encouraged to share preferred educational resources.

### Outcomes Studied

All seven published studies demonstrated either positive-significant, or positive-but-non-significant outcomes in at least some of the measured outcomes (Table 1). No study reported any worsening of outcomes from baseline/pre-intervention. Of the measured outcomes, many were self-reported. Two of the published studies [29], [31] included some form of objective measure – one study [29] used biometric and biochemical markers, another study [31] used an objective test of knowledge. One study [32] conducted a follow-up assessment up to nine months post-intervention.

#### Knowledge and skills

The breadth of knowledge and skill-oriented outcomes included participants' knowledge of their medical condition [29], awareness of what is evidence-based health information [30], [33], knowledge of credible online health resources [31], ability to find reliable online health information [30], ability to find relevant online health information [34], [35], ability to evaluate reliability of online health information [32], [33], and general computer/Internet knowledge and skills [35]. The majority of the knowledge outcomes were based on self-perceived measures of knowledge, with the exception of two studies [29], [31]. Carter *et al.* [29] applied a brief test of diabetes knowledge as part of a survey pre- and post-intervention, whereas Gross *et al.* [31] measured knowledge of credible online health resources via a pre-test and post-test questionnaire based on items covered in the interactive workshop. Two studies tested their participants' skills: one [32] tested participants' ability to evaluate reliability of online health information by instructing participants to rate two pre-selected websites on five dimensions of website quality, as specified by a previous study conducted by Kim *et al.* [36]; while the other [34] investigated participants' ability to find relevant online health information by instructing participants to find answers to two questions on a pre-selected health condition.

#### Attitude and behavior

In one study [30], the key attitude and behavior-oriented outcome were participants' change in the way they search for health information, and in another study [32], information-seeking self-efficacy. In both of these studies, attitudes and behaviors were assessed based on self-perceived measures.

#### Health outcomes

One study [29] indirectly assessed health outcomes in patients with diabetes via measurement of biochemical and biometric markers for both treatment and control groups pre- and post-intervention: mean weight, mean blood pressure, and mean hemoglobin A1c.

### Gray Literature Reports

The interventions, outcomes and brief critique of relevant aspects of the two gray literature reports are summarized in Table 2. Both reports relate to projects aimed to facilitate access to reliable health information for various communities in the United States. The *Access to Resources for Community Health (ARCH)* project [Schneider E. ARCH Evaluation Focus Groups. Boston (USA): Massachusetts General Hospital; 2009] focuses on providing access to online health information, whereas the *Medline in the Mountains* project [Carlson G. NN/LM Quarterly Report. Colorado (USA): Poudre Valley Health System; 2003] focuses on providing access to print and electronic health information resources and services.

**Table 2:** Summary of Interventions, Outcomes, and Brief Critique – Gray Literature Reports.

Studies	Sample characteristics	Intervention	Intervention – Style/Content	Outcome(s)	Critique
ARCH	Four seniors who received ARCH training at a Revere senior center in Revere, Massachusetts.	Group training sessions (and website to assist navigation of reliable health information)	***Style***: A series of group training sessions with the option of one-to-one training if needed. ***Content***: Participants were taught general computer skills relevant to using the Internet, and specific skills for using the ARCH website (http://www2.massgeneral.org/library/arch/arch.asp).	***Other***: Focus groups were conducted to evaluate the outcomes of the ARCH training. Participants reported that the ARCH training was sufficient to facilitate use of the ARCH website. They also felt that ARCH was easy to use to find basic health information relevant to their own needs.	***Strengths***: Participant responses were reported verbatim. ***Weaknesses***: No demographic data collected; arguably small sample size for focus groups – no indication of whether saturation of themes was achieved; qualitative data presented as blocks of quotations by individual focus group participants, instead of presenting common themes explored by the group.
Medline in the Mountains	Patrons of Estes Park, Red Feather Lakes, and Wellington Libraries (rural and geographically isolated communities in Colorado).	Small group training sessions (and website to assist navigation of credible health information databases)	***Style***: A series of small group training sessions with one to two participants per group. ***Content***: Training on how to identify, evaluate and use print and electronic health information resources and services. This training was supplemented with a website that was developed as part of this project (http://redfeather.colibraries.org/online-resources/medline-in-the-mountains.html). The website contains information on how to evaluate health information, as well as how to use MEDLINE, MEDLINE-related and PubMed health databases.	***Knowledge***: Participants were reported to be more aware of the existence of online health information, and have better knowledge about how to find credible health resources. How this outcome was evaluated is unclear. ***Other***: The project's website successfully directed participants to credible health resources. Participants did not need to learn how to conduct searches to find these credible health resources. How these outcomes were evaluated is unclear.	***Strengths***: Training piloted prior to rollout, and re-evaluated and refined quarterly. ***Weaknesses***: Initial needs analysis for training based on survey completed by an arguably small sample size (n = 57); no demographic data collected; unclear sample size of participants who completed a pre-/post-intervention survey; Pre-/post-intervention survey mentioned in report, but no clear link to findings in the report.

The use of group training sessions was common to both projects. The *ARCH* training focused on teaching general computer and Internet skills, and how to use their project's website to find reliable health information. The *Medline in the Mountains* training focused on teaching skills to evaluate reliability of health information, and how to use health information databases; their website appeared to supplement the training, as it hosted materials on how to evaluate reliability of health information, and listed various health information databases.

While both projects were conducted in the United States, the *ARCH* project was conducted in a metropolitan city, while the *Medline in the Mountains* initiative addressed various geographically isolated and rural communities. The *ARCH* project utilized focus groups to evaluate their outcomes, while a pre-/post-intervention survey was utilized in the *Medline in the Mountains* study, albeit with limited data reported.

## Discussion

The seven published studies [29]–[35] included in this review presented a number of substantive design limitations, including small samples and the use of descriptive analysis. Outcomes were predominantly assessed via self-reported pre-post measures, which arguably have greater potential for bias [37]–[39]. The majority of these studies did not describe any validation process for the instruments used. Further, only two studies [29], [32] were randomized-controlled studies. Despite this, four [30], [31], [33], [35] of the five remaining studies assessed either participants' attitudes or outcomes based on pre- and post-intervention measures; this facilitates a more meaningful way to compare the impact of the intervention. Nevertheless, only two [30], [35] of these four studies went beyond the use of descriptive statistics to assess statistical significance of their findings. Thus, the design characteristics, analysis, and perceived overall quality of these studies highlight areas to be addressed in future research. Furthermore, the nature of the studies did not lend themselves to being evaluated systematically.

Our review of the intervention type identified that five [30]–[32], [34], [35] studies used workshops with varying levels of interactivity. Although a didactic approach to helping participants search for online health information was not the sole approach to teaching participants, it was a prominent approach that featured in all of the included studies. Didacticism is a prominent, yet arguably outdated, philosophy in education [40]. Despite vast literature suggesting that student-centered pedagogical approaches may be more effective than didactic education in formal education settings [40]-[42], such approaches do not appear prominent in the education of health consumers, as based on our findings, and it is unclear whether people with limited health literacy respond better to, or prefer, didactic approaches to learning. The concept of patient-centered care - whereby patients have active roles in healthcare, and healthcare is based on the individual patient - is flourishing [43]-[44]. It appears that a didactic approach to developing health consumers' online information-seeking skills may only partially address the issue, given factors such as the recognized importance health consumers' play in day-to-day management of their health, and an era of fast-developing technologies and information abundance. Thus, there appears to be potential to apply various student-centered pedagogical approaches to educating health consumers. Further, it can be argued that a potential contributing factor to the positive results demonstrated by the five workshop-based studies is due to engagement [45] between the workshop trainer and the participants. Thus, reproducibility of results could be problematic.

It is also important to note that, of the included studies, only one study [35] explicitly indicated the use of a particular learning framework – collaborative learning. There is therefore potential for future studies to examine different learning frameworks in the online health literacy space.

All of the seven studies measured varying facets of knowledge, skills or both domains; in a majority, knowledge and skill domains were assessed via measures that were self-reported by participants. While it is important to gain insight into participants' perceptions of the impact of the intervention, the use of objective tests would arguably improve objectivity and minimize potential for bias. Despite the apparent lack of objective tests in many of the included studies, it is important to note that there were no reported worsening of outcomes from baseline/pre-intervention in any of the included studies. This suggests that, while the effectiveness of didactic approaches are yet to be compared with other approaches, current identified interventions do not appear to have negative or detrimental outcomes.

In the two included gray literature reports, a didactic approach to helping participants search for reliable online health information was prominent - similar to the findings from the seven included published studies. The intervention for the first of these studies aimed to general computer and Internet skills, with a view to optimize participants' use of a particular website developed specifically for the project. The second study focused on improving skills to evaluate reliability of health information and use of health information databases. These interventions also bear similarity to some of the included published studies. In terms of evaluation of outcomes, no objective measures were used.

### Limitations

Few studies were deemed eligible for inclusion. It is possible that the use of additional search strings may yield more relevant studies. While the search strings were reviewed by all authors of this review (discipline experts), and advice had been sought from a medical librarian prior to the review, participation in the searches by a medical librarian may have provided a different perspective. While extensive, the gray literature search may have had limited reach in some countries. The finding of few relevant published studies on this topic highlights the potential for further research. Furthermore, their varied application of scientific methods limited our ability to compare them systematically.

### Conclusions

Despite the increasing pervasiveness of, and reliance on, the Internet in the area of health information, we found few reports of interventions to assist health consumers to find reliable health information online. The identified studies measured varying facets of knowledge and/or skills, and commonly reported a didactic approach to training their participants. Outcomes studied were predominantly assessed via self-report by participants, and while arguably subjective, were largely positive and there was no report of worsening of outcomes. As such, there is considerable scope for further research to enhance consumer health literacy in the context of sourcing online information. With the pervasion of mobile technologies and popularity of social media, future research could focus on these developments. Specific interventions could focus on transparent labeling of trustworthy websites – or conversely, ‘blacklists’ of websites deemed biased or otherwise unreliable.

Additionally, as only one identified study explicitly indicated the use of a particular learning framework, there is also potential for further research to explore the use of various learning frameworks in the online health literacy space.
